# Lifetime and 1-Month Prevalence of Mental Disorders in Hebei Province, China: A Community-Based Cross-Sectional Study

**DOI:** 10.3389/fpubh.2021.759160

**Published:** 2021-10-14

**Authors:** Long Sun, Yunshu Zhang, Lijun Cui, Jianfeng Li, Lin Li, Xiuli Sun, Yongqiao Liu, Lili Zhang, Keqing Li

**Affiliations:** ^1^Center for Health Management and Policy Research, School of Public Health, Cheeloo College of Medicine, Shandong University, Jinan, China; ^2^NHC Key Lab of Health Economics and Policy Research, Shandong University, Jinan, China; ^3^Hebei Provincial Mental Health Center, Baoding, China

**Keywords:** mental disorder, prevalence, community resident, cross-sectional study, China

## Abstract

**Background:** The prevalence of mental disorders is an important topic, which has been reported in different countries in the world. In China, some studies were also conducted to get the prevalence of mental disorders at the national level and in some metropolitan cities. However, the prevalence of mental disorders in Chinese underdeveloped provinces has not been reported internationally in recent decades. Due to the discrepancy in the social-economic development of different Chinese provinces, we also have reasons to believe the different prevalence of mental disorders between underdeveloped provinces and other provinces.

**Method:** A community-based cross-sectional study was conducted among community residents aged 18 years and older in Hebei province, China. We screened 20,884 community residents in this study, and a Structured Clinical Interview for Diagnostic and Statistical Manual (DSM-IV) Axis I disorders was used to make the diagnoses of mental disorders.

**Results:** The weighted lifetime prevalence of mental disorders was 15.87% (95% CI 15.38–16.38%), and the 1-month prevalence was 10.79% (95% CI 10.37–11.22%). Anxiety disorder ranked first in the classification of mental disorders for both lifetime (6.56%) and 1-month prevalence (6.25%). The weighted lifetime and 1-month region–gender–age-specific prevalence of mental disorders was also analyzed in this study.

**Conclusions:** Mental disorders have been an important issue in Chinese economically underdeveloped regions, and the prevalence was at a high level compared with the results in the 2000s. There are several serious challenges in the work of Chinese mental disorders, which should be paid more attention to.

## Introduction

Mental disorders have been recognized as an important health problem in the world (1, 2). WHO reported that the burden of mental disorders continued to grow with significant impacts on health and major social, human rights, and economic consequences in all countries of the world (3). As we know, studies about the prevalence of mental disorders are fundamental works for controlling mental disorders, and it can help us to allocate the health resources and set up interventions, which should be explored in all the countries of the world (4, 5).

In the previous decades, the worldwide prevalence of mental disorders has been explored in the World Mental Health Survey (WMHS) (6, 7), and the prevalence of some kinds of mental disorders has been reported, such as social anxiety disorder (8), posttraumatic stress disorder (9), and so on (10, 11). Studies were also conducted in different countries to obtain the prevalence of mental disorders, such as the United State of America (USA) (12, 13), European countries (14), Singapore (15), Finland (16), India (17), and so on (18).

In China, there were also some studies, which explored the prevalence of mental disorders. Michael R Phillips et al. reported the prevalence of mental disorders in four provinces during 2001–2005 (19). Huang et al. reported the prevalence of mental disorders in 2013 (20). There were also some studies conducted in Chinese metropolitan cities (21, 22). All of these studies gave us more instructions for the theories and methods of epidemiological investigation in mental disorders (23). However, the prevalence of mental disorders in Chinese underdeveloped provinces has not been reported internationally in recent years. Due to the discrepancy in social-economic development in different Chinese provinces and the effect of economics on mental disorders (24), we have reasons to believe that the prevalence of mental disorders in the underdeveloped province may be also different.

To fill this gap, we conducted a community-based cross-sectional study to get the lifetime and 1-month prevalence of mental disorders in 2018 in Hebei province, China. Hebei is an economically underdeveloped province located in the middle of China, and the GDP per capita ranked 22nd in all of the 31 provinces of the Chinese mainland in 2018. The findings for this study are not only helpful for us to allocate the mental health resources and set up interventions in Hebei province, but they also can give us implications about the distribution of mental disorders in other Chinese economically underdeveloped regions.

## Method

### Study Design and Sample

Hebei is an economically underdeveloped province located in the middle of China. Between April and August 2016, we conducted a cross-sectional study to get the prevalence of mental disorders among community residents aged 18 years and older in Hebei province. In the current study, we interviewed 20,884 community residents, and 6,769 (32.41%) of them were selected to be diagnosed with mental disorders.

To obtain the prevalence of mental disorders in Hebei province, probability proportionate to size was used to sample for the current study. First, we calculated the sample size using the formula: $N=\frac{Z_{1-\frac{\alpha }{2}}^{2}p\left(1-p\right)}{\delta^{2}};$ where $z_{1-\frac{\alpha }{2}}^{2}$ (level of significance of 95% with two-tailed test) = 1.96; *p* (the prevalence of schizophrenia, one of the lowest prevalence in mental disorders) = 0.50% [referred from a previous cross-sectional epidemiological study in Hebei province (25)]; δ (tolerable error) = 0.001. Then, the calculated minimum sample size was 19,112. Considering a response rate of 80%, we set the sample size as 24,000 in the current study. Second, the number of rural and urban samples was proportional to the region-specific population in each of the cities. Third, we randomly selected 1/3 of the districts and 1/5 of the counties in each city, and one street/town was also randomly selected in each district/county. Fourth, at the community and village level, one to three persons were randomly selected according to the sample size in this area. Finally, residents who have been living in the community/village for more than 6 months were chosen in this study. Then, we ranked these residents by age, and 1/10 of them were systematically selected to be interviewed. For the people who rejected the interview or could not be reached, their neighbors of the same gender and age were interviewed in the current study.

### Interview Procedure

In the current study, the survey was organized in two stages. In the first stage, the subjects were screened by a Chinese version general health questionnaire (GHQ) and other eight questions about the risk factors of mental disorders. The risk factors were as follows: (1) poor physical health status in the recent month; (2) poor mental health status in the recent month; (3) thinking or doing things without control in the recent month; (4) restriction behaviors because of phobias in the recent month; (5) feeling nervous or anxious in the recent year; (6) experiencing troubles due to alcohol in the recent year; (7) seeking help because of psychological or psychiatric problems; (8) hospitalization because of psychological or psychiatric problems. Subjects who gave one or more positive responses among these eight questions were defined as “with risk factors.” For the scores of GHQ, we divided them into three groups (high-risk group, average-risk group, and low-risk group). The high-risk group contained subjects with GHQ scores equal to or higher than 4. The average-risk group contained subjects with GHQ scores equal to 2 or 3. The low-risk group contained subjects with GHQ scores equal to 0 or 1.

**Figure 1:**
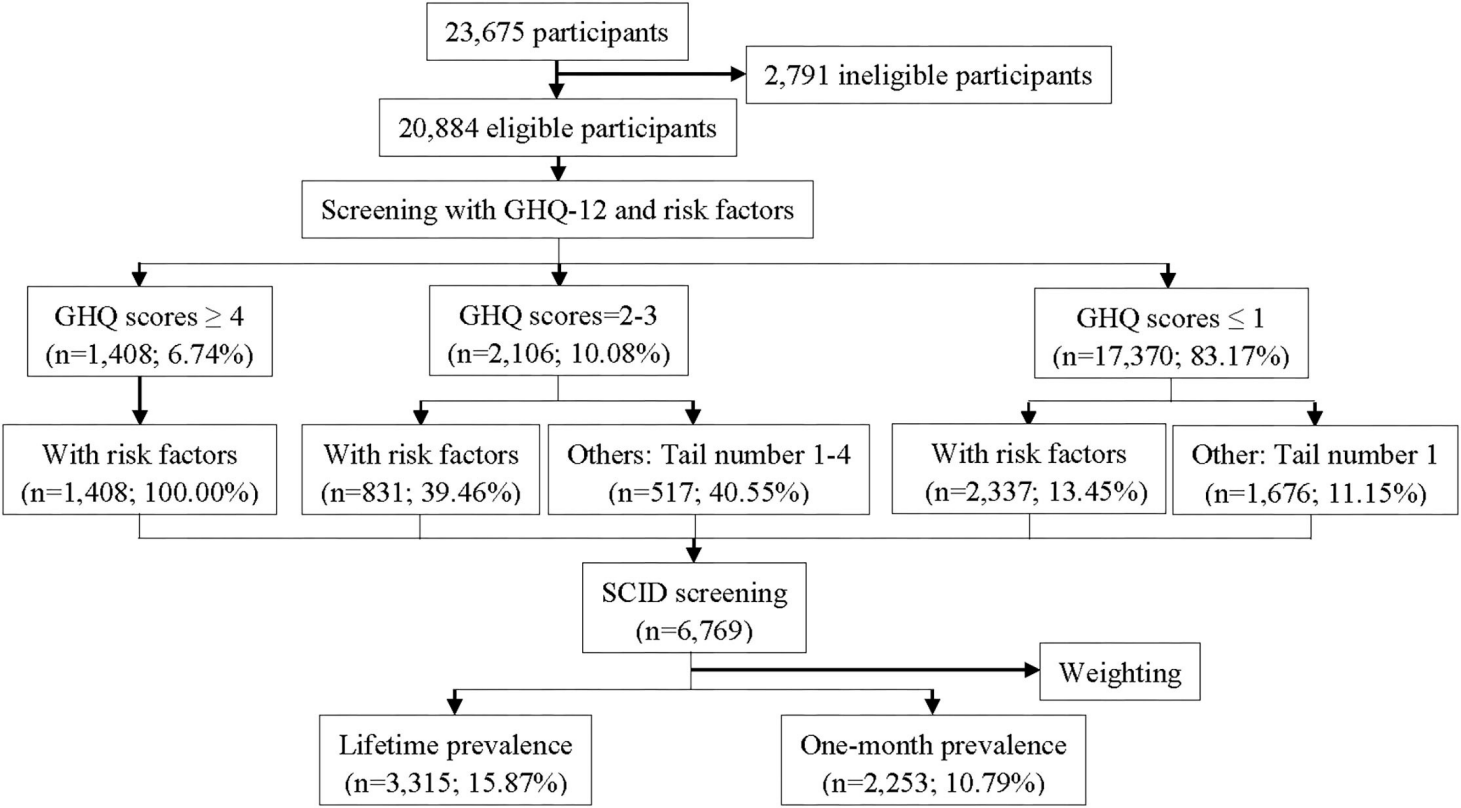
Study flow diagram.

In the second stage, we selected the subjects to be screened by Structured Clinical Interview for Diagnostic and Statistical Manual (DSM-IV) Axis I disorders (SCID) according to the results of GHQ scores and risk factors of mental disorders. First, all subjects with risk factors or in the high-risk group were screened by SCID. Second, subjects in the average-risk group with tail numbers 1–4 were screened by SCID. Third, subjects in the low-risk group with tail number 1 were screened by SCID. A similar selection method was also used in many previous studies (19, 26). The study flow diagram is presented in Figure 1. The Human Research Ethics Committee of Hebei Mental health Center approved the study protocol. Written informed consent was obtained from all the participants in the current study.

### Quality Control

In the whole process of this study, strict quality control was employed to ensure the study quality. First, before the survey, all of the interviewers must be trained for 2 days to make sure they had fully understood the questionnaire and study flow. They were also asked to record the interview. Second, supervisors monitored throughout the survey and randomly checked the interview. Third, SCID diagnoses should be made by psychiatrists with more than 5 years of clinical experience. Fourth, all the questionnaires interviewed in the daytime should be checked again by other interviewers each night. Fifth, subjects with tail number 55 or 66 needed to be retested 5–7 days later by other interviewers in blind. For the subjects with different results, the psychiatrists needed to listen to the interview records and discussed the case to determine the final diagnosis. Finally, double-entry was used to make sure the precision of the data.

### Measures

The Chinese version of the SCID was used to generate the diagnoses of mental disorders (27). The Chinese version of the SCID had been used in many epidemiological studies among Chinese populations, and it was identified with sound reliability and validity (19, 21).

The Chinese version of the General Health Questionnaire (GHQ) was used to select the subjects who were interviewed by SCID (28). This scale also had been used in the Chinese population for many years with good reliability and validity (29–31).

We also collected the gender, age, and region information of the subjects. Gender was indicated by male or female. Age was evaluated by the number of years, and we recorded it into four groups (18–34 years, 35–44 years, 45–59 years, and ≥60 years), which can be seen as an adolescent, young adults, midlife, and elder, respectively. The region was assessed as urban or rural where they lived. All of the subjects should live in the current region for more than 6 months.

### Statistical Methods

IBM SPSS statistics 24.0 for Windows (web edition) and Microsoft Office Excel 2016 for Windows were used for the data analyses. Due to the rules of data collection, we needed to weigh the samples to get the prevalence of mental disorders. The weight used in the current study was generated by three steps (32). In the first step, the subjects with SCID diagnoses (6,769 subjects) were weighted into the number of participants (20,884 subjects) based on the selection of SCID interviews. In the second step, poststratification weights were used to generate the weight of gender–age–region distribution in Hebei province. The distribution of gender, age, and region were based on the data of the 1% population sampling survey in 2015 (33). Finally, we multiplied these two weights to calculate the final weight used for the data analyses.

A chi-square test was applied to compare the significance among different social-demographic populations. The 95% confidence interval for the proportion was calculated by the Wilson score method without continuity correction (34). All tests were two-tailed and a *p* ≤ 0.05 was considered statistically significant.

## Results

In the current study, 23,675 participants were invited to participate in the survey, and 20,884 (88.21%) subjects agreed and completed the questionnaires. With the rules about the scores of GHQ and risk factors of mental disorders, 6,769 (32.41%) of them were selected to diagnose by SCID, and 2,371 (35.03%) subjects were positively diagnosed. Figure 1 was the study flow, which displayed the detailed interview process. We also analyzed the social-demographic information in each stage in Table 1.

**Table 1:** Social-demographic characteristics of the participants (*n* = 20,884).

**Variable**	**Valid sample** **[*n*_**1**_ (*n*_**1**_/*n*)]**	**Valid SCID screening** **[*n*_**2**_ (*n*_**2**_/*n*_**1**_)]**	**Valid SCID diagnoses** **[*n*_**3**_ (*n*_**3**_/*n*_**2**_)]**
Gender			
Male	10,703 (51.25%)	3,004 (28.07%)	1,085 (36.12%)
Female	10,181 (48.75%)	3,765 (36.98%)	1,286 (34.16%)
Age (years old)			
15–34	5,011 (23.99%)	981 (19.58%)	249 (25.38%)
35–44	3,427 (16.41%)	818 (23.87%)	254 (31.05%)
45–59	6,216 (29.76%)	2,251 (36.21%)	867 (38.52%)
≥60	6,230 (29.83%)	2,719 (43.64%)	1,001 (36.82%)
Region			
Urban	6,056 (29.00%)	1,935 (31.95%)	754 (38.97%)
Region	14,828 (71.00%)	4,834 (32.60%)	1,617 (33.45%)
Total	20,884 (100.00%)	6,769 (32.41%)	2,371 (35.03%)

After weighing the sample, we calculated the lifetime and 1-month prevalence of mental disorders. The results showed that the weighted lifetime prevalence of mental disorders was 15.87% (95% CI 15.38–16.38%), and the 1-month prevalence was 10.79% (95% CI 10.37–11.22%). Anxiety disorder ranked first in the classification of mental disorders for both lifetime (6.56%) and 1-month prevalence (6.25%). For lifetime prevalence, the following order was substance use disorder (6.20%), mood disorder (4.58%), psychotic disorders (1.03%), and other mental disorders (0.06%). For 1-month prevalence, the following order was substance use disorder (3.69%), mood disorder (3.06%), psychotic disorders (0.74%), and other mental disorders (0.32%). For the detailed diagnoses, the top five lifetime persons were alcohol use disorders (5.93%), anxiety disorder not otherwise specified (NOS) (3.80%), major depressive disorder (2.93%), specific phobias (1.39%), and depressive disorder NOS (0.99%). Moreover, the top five 1-month disorders were anxiety disorder NOS (3.70%), alcohol use disorders (3.51%), major depressive disorder (1.29%), specific phobias (1.16%), and depressive disorder NOS (0.79%). The detailed lifetime and 1-month prevalence of mental disorders are presented in Table 2.

**Table 2:** Weighted and unweighted lifetime and 1-month prevalence of mental disorders in Hebei province, China (*n* = 20,884).

**Mental disorders**	**Lifetime prevalence**			**1-month prevalence**		
	**Unweighted *n***	**Weighted *n***	**Weighted %** **(95% CI)**	**Unweighted *n***	**Weighted *n***	**Weighted %** **(95% CI)**
Mood disorders	802	957	4.58 (4.31, 4.87)	637	640	3.06 (2.84, 3.31)
Bipolar I disorder	30	32	0.15 (0.11, 0.22)	15	16	0.08 (0.05, 0.12)
Bipolar II disorders	8	8	0.04 (0.02, 0.08)	7	7	0.03 (0.02, 0.07)
Other bipolar disorders	8	15	0.07 (0.04, 0.12)	6	7	0.03 (0.02, 0.07)
Major depressive disorder	499	612	2.93 (2.71, 3.17)	289	270	1.29 (1.15, 1.46)
Dysthymic disorder^*^	–	–	–	135	138	0.66 (0.56, 0.78)
Depressive disorder NOS	165	207	0.99 (0.87, 1.13)	141	164	0.79 (0.67, 0.91)
Mood disorder due to GMC	98	90	0.43 (0.35, 0.53)	77	72	0.34 (0.27, 0.43)
Substance-induced mood disorder	5	5	0.02 (0.01, 0.06)	3	3	0.01 (<0.01, 0.04)
Anxiety disorders	1,043	1,370	6.56 (6.23, 6.90)	1,015	1,306	6.25 (5.93, 6.59)
Panic disorder	88	89	0.43 (0.35, 0.52)	68	72	0.34 (0.27, 0.43)
Agoraphobia without panic	19	21	0.10 (0.07, 0.15)	15	17	0.08 (0.05, 0.13)
Social phobia	19	34	0.16 (0.12, 0.23)	12	10	0.05 (0.03, 0.09)
Specific phobias	177	290	1.39 (1.24, 1.56)	139	242	1.16 (1.02, 1.31)
Obsessive compulsive disorder	29	31	0.15 (0.10, 0.21)	24	21	0.10 (0.07, 0.15)
Post-traumatic stress disorder	118	161	0.77 (0.66, 0.90)	41	58	0.28 (0.21, 0.36)
Generalized anxiety disorder^*^	–	–	–	122	128	0.61 (0.52, 0.73)
Anxiety disorders due to GMC	57	52	0.25 (0.19, 0.33)	54	46	0.22 (0.17, 0.29)
Substance-induced anxiety disorder	6	6	0.03 (0.01, 0.06)	6	6	0.03 (0.01, 0.06)
Anxiety disorder NOS	620	793	3.80 (3.55, 4.06)	607	772	3.70 (3.45, 3.96)
Substance use disorders	621	1,294	6.20 (5.88, 6.53)	400	771	3.69 (3.44, 3.96)
Alcohol use disorders	565	1,239	5.93 (5.62, 6.26)	357	734	3.51 (3.27, 3.77)
Sedative/hypnotic/anxiolytic drug use disorders	51	51	0.24 (0.19, 0.32)	42	37	0.18 (0.13, 0.24)
Other substance use disorders^†^	10	9	0.04 (0.02, 0.08)	7	10	0.05 (0.03, 0.09)
Psychotic disorders	168	215	1.03 (0.90, 1.18)	125	155	0.74 (0.63, 0.87)
Schizophrenia	124	150	0.72 (0.61, 0.84)	99	125	0.60 (0.50, 0.71)
Schizophreniform disorder	2	6	0.03 (0.01, 0.06)	1	5	0.02 (0.01, 0.06)
Schizoaffective disorder	2	2	0.01 (<0.01, 0.03)	2	2	0.01 (<0.01, 0.03)
Delusional disorder	15	15	0.07 (0.04, 0.12)	7	6	0.03 (0.01, 0.06)
Brief psychotic disorder	4	3	0.01 (<0.01, 0.04)	0	0	0.00 (0.00, 0.00)
Psychotic disorder due to GMC	5	5	0.02 (0.01, 0.06)	4	4	0.02 (0.01, 0.05)
Substance-induced psychotic disorder	1	1	<0.01 (<0.01, 0.03)	1	1	<0.01 (<0.01, 0.03)
Psychotic disorder NOS	15	33	0.16 (0.11, 0.22)	11	12	0.06 (0.03, 0.10)
Other mental disorders	4	12	0.06 (0.03, 0.10)	64	66	0.32 (0.25, 0.40)
Somatization disorder^*^	–	–	–	8	9	0.04 (0.02, 0.08)
Pain disorder^*^	–	–	–	32	29	0.14 (0.10, 0.20)
Somatoform disorder NOS^*^	–	–	–	11	9	0.04 (0.02, 0.08)
Hypochondriasis^*^	–	–	–	7	6	0.03 (0.01, 0.06)
Dysmorphic disorder^*^	–	–	–	1	1	<0.01 (<0.01, 0.03)
Adjustment disorder^*^	–	–	–	6	5	0.02 (0.01, 0.06)
Eating disorders^‡^	1	1	<0.01 (<0.01, 0.03)	1	1	<0.01 (<0.01, 0.03)
Other DSM axis I disorders	3	10	0.05 (0.03, 0.09)	3	10	0.05 (0.03, 0.09)
Any mental disorder (excluding dementia)	2,219	3,315	15.87 (15.38, 16.38)	1,881	2,253	10.79 (10.37, 11.22)

*CI, Confidence interval; NOS, Not otherwise specified; GMC, General medical condition.*

* *: Only have a current diagnosis.*

† *: Cannabis, stimulant, opioid, cocaine, hallucinogen, multiple drugs, and other substance use disorders;*

‡ *: Anorexia nervosa, bulimia nervosa, and binge eating disorders*.

We also calculated the gender-specific lifetime and 1-month prevalence of mental disorders. The lifetime and 1-month prevalence of mood disorders and anxiety disorders among women were higher, but the prevalence of substance use disorders was lower. For other mental disorders, the lifetime prevalence was higher in men, but the 1-month prevalence was lower. For men, the top five diagnoses of the weighted lifetime prevalence of mental disorders were alcohol use disorders (11.41%), anxiety disorder NOS (2.77%), major depressive disorder (2.23%), specific phobias (1.00%), and depressive disorder NOS (0.83%). However, for women, posttraumatic stress disorder (1.21%) was in the top five diagnoses instead of alcohol use disorders (0.48%). When we considered the 1-month prevalence among men, the top five were alcohol use disorders (6.80%), anxiety disorder NOS (2.72%), specific phobias (0.82%), major depressive disorder (0.81%), depressive disorder NOS (0.62%) and schizophrenia (0.62%). Nevertheless, for women, dysthymic disorder (1.04%) displaced alcohol use disorders (0.24%) and rose into the top five diagnoses. We also compared the prevalence of each diagnosis between men and women, and most of them were different between men and women (*p* < 0.05). The detailed information was shown in Table 3.

**Table 3:** Weighted lifetime and 1-month prevalence of mental disorders by gender in Hebei province, China (*n* = 20,884).

**Mental disorders**	**Lifetime prevalence**			**1-month prevalence**		
	**Male** **[% (95% CI)]**	**Female** **[% (95% CI)]**	***p***	**Male** **[% (95% CI)]**	**Female** **[% (95% CI)]**	***p***
Mood disorders	3.62 (3.28, 3.99)	5.54 (5.12, 6.00)	<0.001	2.13 (1.87, 2.43)	4.00 (3.64, 4.39)	<0.001
Bipolar I disorder	0.15 (0.09, 0.25)	0.15 (0.09, 0.25)	0.992	0.07 (0.03, 0.14)	0.09 (0.05, 0.16)	0.622
Bipolar II disorders	0.07 (0.03, 0.14)	0.02 (0.01, 0.07)	0.109	0.07 (0.03, 0.14)	0.01 (<0.01, 0.05)	
Other bipolar disorders	0.05 (0.02, 0.11)	0.10 (0.05, 0.18)	0.199	0.01 (0.01, 0.03)	0.02 (0.01, 0.07)	0.288
Major depressive disorder	2.23 (1.96, 2.53)	3.63 (3.29, 4.01)	<0.001	0.81 (0.65, 1.00)	1.78 (1.54, 2.05)	<0.001
Dysthymic disorder^*^	–	–	–	0.28 (0.19, 0.40)	1.04 (0.86, 1.26)	<0.001
Depressive disorder NOS	0.83 (0.67, 1.02)	1.16 (0.97, 1.38)	0.016	0.62 (0.49, 0.79)	0.94 (0.77, 1.14)	0.010
Mood disorder due to GMC	0.31 (0.22, 0.43)	0.55 (0.43, 0.72)	0.006	0.30 (0.21, 0.42)	0.39 (0.29, 0.53)	0.244
Substance-induced mood disorder	0.04 (0.01, 0.10)	0.01 (<0.01, 0.05)	0.178	0.03 (0.01, 0.08)	0.00 (0.00, 0.00)	0.124
Anxiety disorders	4.62 (4.24, 5.04)	8.49 (7.97, 9.04)	<0.001	4.46 (4.08, 4.87)	8.04 (7.53, 8.58)	<0.001
Panic disorder	0.16 (0.10, 0.26)	0.69 (0.55, 0.87)	<0.001	0.12 (0.07, 0.21)	0.56 (0.44, 0.73)	<0.001
Agoraphobia without panic	0.02 (0.01, 0.07)	0.19 (0.12, 0.30)	<0.001	0.02 (0.01, 0.07)	0.15 (0.09, 0.25)	0.001
Social phobia	0.22 (0.15, 0.33)	0.11 (0.06, 0.19)	0.038	0.03 (0.01, 0.08)	0.07 (0.03, 0.14)	0.344
Specific phobias	1.00 (0.82, 1.21)	1.78 (1.54, 2.05)	<0.001	0.82 (0.66, 1.01)	1.51 (1.29, 1.76)	<0.001
Obsessive compulsive disorder	0.13 (0.08, 0.23)	0.16 (0.10, 0.26)	0.597	0.06 (0.03, 0.13)	0.14 (0.09, 0.24)	0.050
Post-traumatic stress disorder	0.32 (0.23, 0.44)	1.21 (1.02, 1.44)	<0.001	0.08 (0.04, 0.15)	0.49 (0.37, 0.64)	<0.001
Generalized anxiety disorder^*^	–	–	–	0.57 (0.44, 0.73)	0.66 (0.52, 0.83)	0.387
Anxiety disorders due to GMC	0.19 (0.12, 0.30)	0.31 (0.22, 0.43)	0.098	0.13 (0.08, 0.23)	0.31 (0.22, 0.43)	0.008
Substance-induced anxiety disorder	0.04 (0.01, 0.10)	0.02 (0.01, 0.07)	0.412	0.04 (0.01, 0.10)	0.02 (0.01, 0.07)	0.452
Anxiety disorder NOS	2.77 (2.47, 3.11)	4.83 (4.43, 5.26)	<0.001	2.72 (2.43, 3.06)	4.66 (4.28, 5.09)	<0.001
Substance use disorders	11.61 (11.01, 12.24)	0.79 (0.64, 0.98)	<0.001	6.96 (6.48, 7.46)	0.44 (0.33, 0.59)	<0.001
Alcohol use disorders	11.41 (10.81, 12.03)	0.48 (0.36, 0.63)	<0.001	6.80 (6.33, 7.30)	0.24 (0.16, 0.35)	<0.001
Sedative/hypnotic/anxiolytic drug use disorders	0.23 (0.15, 0.34)	0.26 (0.18, 0.38)	0.684	0.17 (0.11, 0.27)	0.18 (0.12, 0.28)	0.878
Other substance use disorders^†^	0.02 (0.01, 0.07)	0.07 (0.03, 0.14)	0.180	0.07 (0.03, 0.14)	0.03 (0.01, 0.08)	0.226
Psychotic disorders	1.07 (0.89, 1.29)	0.98 (0.81, 1.19)	0.520	0.78 (0.63, 0.96)	0.71 (0.56, 0.89)	0.557
Schizophrenia	0.71 (0.57, 0.89)	0.74 (0.59, 0.92)	0.823	0.62 (0.49, 0.79)	0.57 (0.45, 0.74)	0.639
Schizophreniform disorder	0.06 (0.03, 0.13)	0.00 (0.00, 0.00)	0.014	0.05 (0.02, 0.11)	0.00 (0.00, 0.00)	0.031
Schizoaffective disorder	0.00 (0.00, 0.00)	0.02 (0.01, 0.07)	0.500	0.00 (0.00, 0.00)	0.02 (0.01, 0.07)	0.500
Delusional disorder	0.03 (0.01, 0.08)	0.11 (0.07, 0.20)	0.020	0.01 (<0.01, 0.05)	0.06 (0.03, 0.13)	0.125
Brief psychotic disorder	0.02 (0.01, 0.07)	0.02 (0.01, 0.07)	1.000	0.00 (0.00, 0.00)	0.00 (0.00, 0.00)	–
Psychotic disorder due to GMC	0.03 (0.01, 0.08)	0.02 (0.01, 0.07)	0.687	0.03 (0.01, 0.08)	0.01 (<0.01, 0.05)	0.374
Substance-induced psychotic disorder	0.00 (0.00, 0.00)	0.01 (<0.01, 0.05)	1.000	0.00 (0.00, 0.00)	0.01 (<0.01, 0.05)	1.000
Psychotic disorder NOS	0.24 (0.16, 0.35)	0.09 (0.05, 0.16)	0.006	0.07 (0.03, 0.14)	0.05 (0.02, 0.11)	0.559
Other mental disorders	0.09 (0.05, 0.16)	0.02 (0.01, 0.07)	0.034	0.21 (0.14, 0.32)	0.42 (0.31, 0.56)	0.007
Somatization disorder^*^	–	–	–	0.01 (<0.01, 0.05)	0.08 (0.04, 0.15)	0.039
Pain disorder^*^	–	–	–	0.08 (0.04, 0.15)	0.21 (0.14, 0.32)	0.011
Somatoform disorder NOS^*^	–	–	–	0.02 (0.01, 0.07)	0.07 (0.03, 0.14)	0.180
Hypochondriasis^*^	–	–	–	0.02 (0.01, 0.07)	0.04 (0.01, 0.10)	0.687
Dysmorphic disorder^*^	–	–	–	0.00 (0.00, 0.00)	0.01 (<0.01, 0.05)	1.000
Adjustment disorder^*^	–	–	–	0.01 (<0.01, 0.05)	0.04 (0.01, 0.10)	0.375
Eating disorders^‡^	0.00 (0.00, 0.00)	0.01 (<0.01, 0.05)	0.318	0.00 (0.00, 0.00)	0.01 (<0.01, 0.05)	1.000
Other DSM axis I disorders	0.09 (0.05, 0.16)	0.01 (<0.01, 0.05)	0.011	0.09 (0.05, 0.16)	0.01 (<0.01, 0.05)	0.012
Any mental disorder (excluding dementia)	18.13 (17.41, 18.88)	13.62 (12.98, 14.29)	<0.001	12.72 (12.10, 13.38)	11.73 (11.13, 12.36)	0.029

*CI, Confidence interval; NOS, Not otherwise specified; GMC, General medical condition.*

* *: Only have a current diagnosis.*

† *: Cannabis, stimulant, opioid, cocaine, hallucinogen, multiple drugs, and other substance use disorders;*

‡ *: Anorexia nervosa, bulimia nervosa, and binge eating disorders*.

In Tables 4, 5, we analyzed the weighted lifetime and 1-month age-specific prevalence of mental disorders, respectively. We also compared the prevalence among the different age groups in these two tables. Generally, both lifetime and 1-month prevalence of mood disorders and anxiety disorders increased with age. For substance use disorder, the lifetime and 1-month prevalence also increased with the age, with an exception of elders (aged ≥60 years).For people aged 18–34 years and 35–44 years, the top five diagnoses of the weighted lifetime prevalence were alcohol use disorders (4.43%, 4.90%), anxiety disorder NOS (2.44%, 3.56%), specific phobias (1.24%, 0.71%), major depressive disorder (1.21%, 1.70%), and schizophrenia (0.51%, 1.23%). For people aged 45–59 years and ≥60 years, depressive disorder NOS replaced schizophrenia and rose into the top five diagnoses. When we conducted to the weighted 1-month prevalence of mental disorders, the top five diagnoses for people aged 18–34 years were alcohol use disorders (2.62%), anxiety disorder NOS (2.37%), specific phobias (1.13%), schizophrenia (0.44%), and depressive disorder NOS (0.38%). For people aged 35–44 years, the order changed into alcohol use disorders (3.53%), anxiety disorder NOS (3.48%), schizophrenia (1.01%), major depressive disorder (0.68%), specific phobias (0.63%), and depressive disorder NOS (0.63%). For people aged 45–59 years, the order was alcohol use disorders (4.45%), anxiety disorder NOS (4.15%), major depressive disorder (1.93%), specific phobias (1.15%), and depressive disorder NOS (1.03%). For elders (≥60 years), the order was anxiety disorder NOS (5.34%), alcohol use disorders (3.61%), major depressive disorder (2.44%), Specific phobias (1.69%), and dysthymic disorder (1.32%).

**Table 4:** Weighted lifetime prevalence of mental disorders by age in Hebei province, China (*n* = 20,884).

**Mental disorders**	**18–34years** **[% (95% CI)]**	**35–44 years** **[% (95% CI)]**	**45–59 years** **[% (95% CI)]**	**≥60 years** **[% (95% CI)]**	***p ***
Mood disorders	1.86 (1.56, 2.20)	2.71 (2.23, 3.29)	6.54 (5.95, 7.20)	7.73 (6.98, 8.56)	<0.001
Bipolar I disorder	0.09 (0.04, 0.19)	0.14 (0.06, 0.32)	0.18 (0.10, 0.33)	0.23 (0.12, 0.42)	0.267
Bipolar II disorders	0.03 (0.01, 0.11)	0.00 (0.00, 0.00)	0.08 (0.04, 0.19)	0.05 (0.01, 0.17)	0.247
Other bipolar disorders	0.04 (0.01, 0.13)	0.03 (<0.01, 0.15)	0.05 (0.02, 0.15)	0.21 (0.11, 0.39)	0.007
Major depressive disorder	1.21 (0.98, 1.50)	1.70 (1.33, 2.17)	4.20 (3.72, 4.73)	4.91 (4.31, 5.59)	<0.001
Depressive disorder NOS	0.39 (0.27, 0.57)	0.66 (0.44, 0.98)	1.40 (1.13, 1.73)	1.62 (1.29, 2.04)	<0.001
Mood disorder due to GMC	0.13 (0.07, 0.25)	0.14 (0.06, 0.32)	0.70 (0.52, 0.94)	0.78 (0.56, 1.08)	<0.001
Substance-induced mood disorder	0.01 (<0.01, 0.08)	0.08 (0.03, 0.24)	0.00 (0.00, 0.00)	0.02 (<0.01, 0.13)	0.097
Anxiety disorders	4.21 (3.76, 4.71)	4.52 (3.89, 5.24)	7.93 (7.27, 8.64)	10.13 (9.27, 11.06)	<0.001
Panic disorder	0.06 (0.02, 0.15)	0.03(<0.01, 0.15)	0.63 (0.46, 0.87)	1.07 (0.81, 1.42)	<0.001
Agoraphobia without panic	0.04 (0.01, 0.13)	0.05 (0.02, 0.20)	0.12 (0.06, 0.24)	0.21 (0.11, 0.39)	0.057
Social phobia	0.28 (0.18, 0.43)	0.14 (0.06, 0.32)	0.08 (0.04, 0.19)	0.09 (0.04, 0.23)	0.022
Specific phobias	1.24 (1.01, 1.53)	0.71 (0.49, 1.04)	1.65 (1.36, 2.00)	1.83 (1.47, 2.27)	<0.001
Obsessive compulsive disorder	0.09 (0.04, 0.19)	0.00 (0.00, 0.00)	0.18 (0.10, 0.33)	0.32 (0.19, 0.54)	0.001
Post-traumatic stress disorder	0.32 (0.21, 0.49)	0.22 (0.11, 0.43)	1.05 (0.82, 1.34)	1.55 (1.23, 1.96)	<0.001
Anxiety disorders due to GMC	0.04 (0.01, 0.13)	0.08 (0.03, 0.24)	0.38 (0.26, 0.57)	0.52 (0.35, 0.79)	<0.001
Substance-induced anxiety disorder	0.00 (0.00, 0.00)	0.03 (<0.01, 0.15)	0.05 (0.02, 0.15)	0.05 (0.01, 0.17)	0.166
Anxiety disorder NOS	2.44 (2.10, 2.83)	3.56 (3.00, 4.21)	4.33 (3.84, 4.88)	5.41 (4.78, 6.12)	<0.001
Substance use disorders	4.44 (3.98, 4.96)	5.01 (4.35, 5.77)	7.96 (7.30, 8.67)	7.48 (6.74, 8.30)	<0.001
Alcohol use disorders	4.43 (3.97, 4.94)	4.90 (4.25, 5.65)	7.73 (7.08, 8.43)	6.66 (5.96, 7.44)	<0.001
Sedative/hypnotic/anxiolytic drug use disorders	0.00 (0.00, 0.00)	0.08 (0.03, 0.24)	0.25 (0.15, 0.41)	0.75 (0.54, 1.06)	<0.001
Other substance use disorders^†^	0.01 (<0.01, 0.08)	0.03 (<0.01, 0.15)	0.05 (0.02, 0.15)	0.09 (0.04, 0.23)	0.282
Psychotic disorders	0.95 (0.75, 1.21)	1.42 (1.09, 1.86)	0.92 (0.70, 1.19)	0.96 (0.71, 1.29)	0.072
Schizophrenia	0.51 (0.37, 0.71)	1.23 (0.92, 1.64)	0.63 (0.46, 0.87)	0.73 (0.52, 1.03)	<0.001
Schizophreniform disorder	0.07 (0.03, 0.17)	0.00 (0.00, 0.00)	0.00 (0.00, 0.00)	0.02 (<0.01, 0.13)	0.057
Schizoaffective disorder	0.00 (0.00, 0.00)	0.00 (0.00, 0.00)	0.02 (<0.01, 0.09)	0.02 (<0.01, 0.13)	0.416
Delusional disorder	0.01 (<0.01, 0.08)	0.11 (0.04, 0.28)	0.10 (0.05, 0.22)	0.07 (0.02, 0.20)	0.190
Brief psychotic disorder	0.00 (0.00, 0.00)	0.03 (<0.01, 0.15)	0.02 (<0.01, 0.09)	0.05 (0.01, 0.17)	0.272
Psychotic disorder due to GMC	0.04 (0.01, 0.13)	0.00 (0.00, 0.00)	0.02 (<0.01, 0.09)	0.02 (<0.01, 0.13)	0.422
Substance-induced psychotic disorder	0.00 (0.00, 0.00)	0.00 (0.00, 0.00)	0.02 (<0.01, 0.09)	0.00 (0.00, 0.00)	0.477
Psychotic disorder NOS	0.31 (0.20, 0.47)	0.03 (<0.01, 0.15)	0.15(0.08, 0.28)	0.07 (0.02, 0.20)	0.002
Other mental disorders	0.01 (<0.01, 0.08)	0.00 (0.00, 0.00)	0.15 (0.08, 0.28)	0.02 (<0.01, 0.13)	0.002
Eating disorders^‡^	0.01 (<0.01, 0.08)	0.00 (0.00, 0.00)	0.00 (0.00, 0.00)	0.00 (0.00, 0.00)	0.526
Other DSM axis I disorders	0.00 (0.00, 0.00)	0.00 (0.00, 0.00)	0.15 (0.08, 0.28)	0.02 (<0.01, 0.13)	<0.001
Any mental disorder r(excluding dementia)	10.36 (9.66, 11.10)	12.13 (11.11, 13.23)	20.03 (19.04, 21.06)	21.90 (20.70, 23.15)	<0.001

*CI, Confidence interval; NOS, Not otherwise specified; GMC, General medical condition.*

† *: Cannabis, stimulant, opioid, cocaine, hallucinogen, multiple drugs, and other substance use disorders;*

‡ *: Anorexia nervosa, bulimia nervosa, and binge eating disorders*.

**Table 5:** Weighted 1-month prevalence of mental disorders by age in Hebei province, China (*n*=20,884).

**Mental disorders**	**18–34 years** **[% (95% CI)]**	**35–44 years** **[% (95% CI)]**	**45–59 years** **[% (95% CI)]**	**≥60 years** **[% (95% CI)]**	***p***
Mood disorders	1.07 (0.85, 1.34)	1.83 (1.45, 2.32)	4.30 (3.81, 4.84)	5.52 (4.88, 6.24)	<0.001
Bipolar I disorder	0.06 (0.02, 0.15)	0.05 (0.02, 0.20)	0.08 (0.04, 0.19)	0.11 (0.05, 0.27)	0.728
Bipolar II disorders	0.03 (0.01, 0.11)	0.00 (0.00, 0.00)	0.07 (0.03, 0.17)	0.05 (0.01, 0.17)	0.257
Other bipolar disorders	0.04 (0.01, 0.13)	0.03 (<0.01, 0.15)	0.03 (0.01, 0.12)	0.05 (0.01, 0.17)	0.964
Major depressive disorder	0.32 (0.21, 0.49)	0.68 (0.46, 1.01)	1.93 (1.61, 2.31)	2.44 (2.02, 2.94)	<0.001
Dysthymic disorder	0.12 (0.06, 0.23)	0.41 (0.25, 0.68)	0.93 (0.72, 1.21)	1.32 (1.03, 1.71)	<0.001
Depressive disorder NOS	0.38 (0.26, 0.56)	0.63 (0.42, 0.94)	1.03 (0.81, 1.32)	1.21 (0.93, 1.58)	<0.001
Mood disorder due to GMC	0.12 (0.06, 0.23)	0.03 (<0.01, 0.15)	0.53 (0.38, 0.75)	0.68 (0.48, 0.98)	<0.001
Substance-induced mood disorder	0.00 (0.00, 0.00)	0.03 (<0.01, 0.15)	0.00 (0.00, 0.00)	0.02 (<0.01, 0.13)	0.280
Anxiety disorders	3.97 (3.54, 4.46)	4.46 (3.84, 5.18)	7.36 (6.73, 8.05)	9.79 (8.94, 10.70)	<0.001
Panic disorder	0.06 (0.02, 0.15)	0.00 (0.00, 0.00)	0.43 (0.30, 0.63)	0.96 (0.71, 1.29)	<0.001
Agoraphobia without panic	0.01 (<0.01, 0.08)	0.05 (0.02, 0.20)	0.10 (0.05, 0.22)	0.18 (0.09, 0.36)	0.016
Social phobia	0.00 (0.00, 0.00)	0.05 (0.02, 0.20)	0.07 (0.03, 0.17)	0.09 (0.04, 0.23)	0.039
Specific phobias	1.13 (0.90, 1.40)	0.63 (0.42, 0.94)	1.15 (0.91, 1.45)	1.69 (1.35, 2.11)	<0.001
Obsessive compulsive disorder	0.09 (0.04, 0.19)	0.00 (0.00, 0.00)	0.15 (0.08, 0.28)	0.14 (0.06, 0.30)	0.026
Post-traumatic stress disorder	0.12 (0.06, 0.23)	0.11 (0.04, 0.28)	0.35 (0.23, 0.53)	0.59 (0.41, 0.87)	<0.001
Generalized anxiety disorder	0.26 (0.17, 0.42)	0.22 (0.11, 0.43)	0.93 (0.72, 1.21)	1.07 (0.81, 1.42)	<0.001
Anxiety disorders due to GMC	0.04 (0.01, 0.13)	0.08 (0.03, 0.24)	0.38 (0.26, 0.57)	0.39 (0.24, 0.62)	<0.001
Substance-induced anxiety disorder	0.00 (0.00, 0.00)	0.03 (<0.01, 0.15)	0.05 (0.02, 0.15)	0.05 (0.01, 0.17)	0.166
Anxiety disorder NOS	2.37 (2.03, 2.75)	3.48 (2.93, 4.12)	4.15 (3.67, 4.68)	5.34 (4.71, 6.05)	<0.001
Substance use disorders	2.62 (2.26, 3.02)	3.61 (3.05, 4.27)	4.60 (4.10, 5.16)	4.20 (3.64, 4.83)	<0.001
Alcohol use disorders	2.62 (2.26, 3.02)	3.53 (2.98, 4.18)	4.45 (3.95, 5.00)	3.61 (3.09, 4.20)	<0.001
Sedative/hypnotic/anxiolytic drug use disorders	0.00 (0.00, 0.00)	0.08 (0.03, 0.24)	0.18 (0.10, 0.33)	0.52 (0.35, 0.79)	<0.001
Other substance use disorders^†^	0.07 (0.03, 0.17)	0.00 (0.00, 0.00)	0.02 (<0.01, 0.09)	0.09 (0.04, 0.23)	0.064
Psychotic disorders	0.60 (0.44, 0.81)	1.04 (0.76, 1.42)	0.70 (0.52, 0.94)	0.78 (0.56, 1.08)	0.089
Schizophrenia	0.44 (0.31, 0.63)	1.01 (0.74, 1.39)	0.53 (0.38, 0.75)	0.59 (0.41, 0.87)	0.003
Schizophreniform disorder	0.07 (0.03, 0.17)	0.00 (0.00, 0.00)	0.00 (0.00, 0.00)	0.00 (0.00, 0.00)	0.011
Schizoaffective disorder	0.00 (0.00, 0.00)	0.00 (0.00, 0.00)	0.02 (<0.01, 0.09)	0.02 (<0.01, 0.13)	0.416
Delusional disorder	0.01 (<0.01, 0.08)	0.00 (0.00, 0.00)	0.03 (0.01, 0.12)	0.07 (0.02, 0.20)	0.217
Brief psychotic disorder	0.00 (0.00, 0.00)	0.00 (0.00, 0.00)	0.00 (0.00, 0.00)	0.00 (0.00, 0.00)	1.000
Psychotic disorder due to GMC	0.03 (0.01, 0.11)	0.00 (0.00, 0.00)	0.02 (<0.01, 0.09)	0.02 (<0.01, 0.13)	0.624
Substance-induced psychotic disorder	0.00 (0.00, 0.00)	0.00 (0.00, 0.00)	0.02 (<0.01, 0.09)	0.00 (0.00, 0.00)	0.477
Psychotic disorder NOS	0.04 (0.01, 0.13)	0.03 (<0.01, 0.15)	0.08 (0.04, 0.19)	0.07 (0.02, 0.20)	0.647
Other mental disorders	0.01 (<0.01, 0.08)	0.16 (0.08, 0.36)	0.53 (0.38, 0.75)	0.82 (0.59, 1.14)	<0.001
Somatization disorder	0.00 (0.00, 0.00)	0.03 (<0.01, 0.15)	0.10 (0.05, 0.22)	0.05 (0.01, 0.17)	0.024
Pain disorder	0.00 (0.00, 0.00)	0.05 (0.02, 0.20)	0.23 (0.14, 0.39)	0.30 (0.17, 0.51)	<0.001
Somatoform disorder NOS	0.00 (0.00, 0.00)	0.08 (0.03, 0.24)	0.02 (<0.01, 0.09)	0.14 (0.06, 0.30)	0.003
Hypochondriasis	0.00 (0.00, 0.00)	0.00 (0.00, 0.00)	0.05 (0.02, 0.15)	0.07 (0.02, 0.20)	0.036
Dysmorphic disorder	0.00 (0.00, 0.00)	0.00 (0.00, 0.00)	0.02 (<0.01, 0.09)	0.00 (0.00, 0.00)	0.477
Adjustment disorder	0.00 (0.00, 0.00)	0.00 (0.00, 0.00)	0.02 (<0.01, 0.09)	0.09 (0.04, 0.23)	0.019
Eating disorders^‡^	0.01 (<0.01, 0.08)	0.00 (0.00, 0.00)	0.00 (0.00, 0.00)	0.00 (0.00, 0.00)	0.526
Other DSM axis I disorders	0.00 (0.00, 0.00)	0.00 (0.00, 0.00)	0.15 (0.08, 0.28)	0.02 (<0.01, 0.13)	<0.001
Any mental disorder (excluding dementia)	7.86 (7.25, 8.52)	9.72 (8.80, 10.72)	14.82 (13.95, 15.74)	17.57 (16.47, 18.72)	<0.001

*CI, Confidence interval; NOS, Not otherwise specified; GMC, General medical condition.*

† *: Cannabis, stimulant, opioid, cocaine, hallucinogen, multiple drugs, and other substance use disorders;*

‡ *: Anorexia nervosa, bulimia nervosa, and binge eating disorders*.

Region-specific prevalence is shown in Table 6. We also compared the different prevalence between rural and urban regions. All of the classifications of mental disorders were statistically significant (*p* <0.05) between the rural and urban regions, apart from other mental disorders. The weighted lifetime and 1-month prevalence of mood disorders and psychotic disorders were higher in the rural region, but the prevalence of anxiety disorders and substance use disorders was lower. Alcohol use disorders, anxiety disorder NOS, major depressive disorder, specific phobias, depressive disorder NOS were the top five diagnoses in the urban and rural region.

**Table 6:** Weighted lifetime and 1-month prevalence of mental disorders by region in Hebei province, China (*n*=20,884).

**Mental disorders**	**Lifetime prevalence**			**1-month prevalence**		
	**Urban** **[% (95% CI)]**	**Rural** **[% (95% CI)]**	***p***	**Urban** **[% (95% CI)]**	**Rural** **[% (95% CI)]**	***p***
Mood disorders	3.93 (3.42, 4.52)	4.78 (4.46, 5.12)	0.014	2.36 (1.97, 2.83)	3.27 (3.01, 3.56)	0.001
Bipolar I disorder	0.17 (0.08, 0.33)	0.15 (0.10, 0.22)	0.776	0.04 (0.01, 0.15)	0.09 (0.05, 0.15)	0.550
Bipolar II disorders	0.00 (0.00, 0.00)	0.05 (0.03, 0.10)	0.123	0.00 (0.00, 0.00)	0.04 (0.02, 0.09)	0.363
Other bipolar disorders	0.06 (0.02, 0.18)	0.07 (0.04, 0.13)	0.790	0.06 (0.02, 0.18)	0.02 (0.01, 0.05)	0.137
Major depressive disorder	2.51 (2.10, 2.99)	3.06 (2.81, 3.34)	0.047	0.73 (0.53, 1.02)	1.46 (1.29, 1.66)	<0.001
Dysthymic disorder^*^	–	–	–	0.52 (0.35, 0.77)	0.70 (0.58, 0.84)	0.195
Depressive disorder NOS	0.84 (0.62, 1.14)	1.04 (0.89, 1.21)	0.220	0.75 (0.54, 1.04)	0.79 (0.67, 0.94)	0.774
Mood disorder due to GMC	0.36 (0.22, 0.57)	0.45 (0.36, 0.57)	0.365	0.29 (0.17, 0.49)	0.36 (0.28, 0.47)	0.486
Substance-induced mood disorder	0.02 (<0.01, 0.12)	0.03 (0.01, 0.07)	0.717	0.02 (<0.01, 0.12)	0.01 (<0.01, 0.05)	0.542
Anxiety disorders	7.91 (7.17, 8.71)	6.17 (5.81, 6.55)	<0.001	7.80 (7.07, 8.60)	5.80 (5.45, 6.17)	<0.001
Panic disorder	0.31 (0.19, 0.52)	0.46 (0.37, 0.58)	0.174	0.19 (0.10, 0.36)	0.39 (0.30, 0.49)	0.040
Agoraphobia without panic	0.15 (0.07, 0.30)	0.09 (0.06, 0.15)	0.319	0.13 (0.06, 0.27)	0.07 (0.04, 0.12)	0.247
Social phobia	0.06 (0.02, 0.18)	0.19 (0.14, 0.27)	0.051	0.04 (0.01, 0.15)	0.05 (0.03, 0.10)	1.000
Specific phobias	1.38 (1.09, 1.75)	1.39 (1.22, 1.58)	0.956	0.96 (0.72, 1.28)	1.22 (1.06, 1.41)	0.139
Obsessive compulsive disorder	0.17 (0.08, 0.33)	0.14 (0.10, 0.21)	0.699	0.15 (0.07, 0.30)	0.09 (0.05, 0.15)	0.296
Post-traumatic stress disorder	0.23 (0.13, 0.41)	0.93 (0.79, 1.09)	<0.001	0.06 (0.02, 0.18)	0.34 (0.26, 0.44)	0.001
Generalized anxiety disorder^*^	–	–	–	0.67 (0.47, 0.94)	0.60 (0.49, 0.73)	0.604
Anxiety disorders due to GMC	0.19 (0.10, 0.36)	0.27 (0.20, 0.36)	0.338	0.08 (0.03, 0.21)	0.26 (0.19, 0.35)	0.022
Substance-induced anxiety disorder	0.00 (0.00, 0.00)	0.04 (0.02, 0.08)	0.348	0.00 (0.00, 0.00)	0.04 (0.02, 0.08)	0.348
Anxiety disorder NOS	5.92 (5.29, 6.62)	3.17 (2.91, 3.46)	<0.001	5.79 (5.17, 6.49)	3.07 (2.82, 3.35)	<0.001
Substance use disorders	8.47 (7.71, 9.29)	5.52 (5.18, 5.88)	<0.001	4.12 (3.59, 4.72)	3.56 (3.28, 3.86)	0.070
Alcohol use disorders	8.03 (7.30, 8.84)	5.30 (4.97, 5.66)	<0.001	3.91 (3.40, 4.50)	3.40 (3.13, 3.69)	0.090
Sedative/hypnotic/anxiolytic drug use disorders	0.42 (0.27, 0.65)	0.19 (0.13, 0.27)	0.006	0.25 (0.14, 0.44)	0.16 (0.11, 0.23)	0.167
Other substance use disorders^†^	0.02 (<0.01, 0.12)	0.05 (0.03, 0.10)	0.694	0.00 (0.00, 0.00)	0.06 (0.03, 0.11)	0.130
Psychotic disorders	0.59 (0.41, 0.85)	1.16 (1.01, 1.34)	0.001	0.48 (0.32, 0.72)	0.82 (0.69, 0.97)	0.017
Schizophrenia	0.44 (0.29, 0.67)	0.80 (0.67, 0.95)	0.009	0.40 (0.25, 0.62)	0.66 (0.54, 0.80)	0.040
Schizophreniform disorder	0.00 (0.00, 0.00)	0.04 (0.02, 0.08)	0.348	0.00 (0.00, 0.00)	0.03 (0.01, 0.07)	0.595
Schizoaffective disorder	0.00 (0.00, 0.00)	0.01 (<0.01, 0.05)	1.000	0.00 (0.00, 0.00)	0.01 (<0.01, 0.05)	1.000
Delusional disorder	0.00 (0.00, 0.00)	0.09 (0.06, 0.15)	0.030	0.00 (0.00, 0.00)	0.04 (0.02, 0.08)	0.348
Brief psychotic disorder	0.02 (<0.01, 0.12)	0.02 (0.01, 0.05)	1.000	0.00 (0.00, 0.00)	0.00 (0.00, 0.00)	1.000
Psychotic disorder due to GMC	0.06 (0.02, 0.18)	0.01 (<0.01, 0.05)	0.082	0.04 (0.01, 0.15)	0.01 (<0.01, 0.05)	0.227
Substance-induced psychotic disorder	0.00 (0.00, 0.00)	0.01 (<0.01, 0.04)	1.000	0.06 (0.03, 0.11)	0.01 (<0.01, 0.04)	1.000
Psychotic disorder NOS	0.06 (0.02, 0.18)	0.19 (0.13, 0.27)	0.059	0.06 (0.02, 0.18)	0.06 (0.03, 0.11)	1.000
Other mental disorders	0.04 (0.01, 0.15)	0.06 (0.03, 0.11)	1.000	0.27 (0.16, 0.46)	0.33 (0.25, 0.43)	0.536
Somatization disorder^*^	–	–	–	0.00 (0.00, 0.00)	0.06 (0.03, 0.11)	0.225
Pain disorder^*^	–	–	–	0.15 (0.07, 0.30)	0.14 (0.09, 0.21)	0.827
Somatoform disorder NOS^*^	–	–	–	0.08 (0.03, 0.21)	0.03 (0.01, 0.07)	0.129
Hypochondriasis^*^	–	–	–	0.00 (0.00, 0.00)	0.03 (0.01, 0.07)	0.595
Dysmorphic disorder^*^	–	–	–	0.00 (0.00, 0.00)	0.01 (<0.01, 0.04)	1.000
Adjustment disorder^*^	–	–	–	0.00 (0.00, 0.00)	0.03 (0.01, 0.07)	0.595
Eating disorders^‡^	0.02 (<0.01, 0.12)	0.00 (0.00, 0.00)	0.229	0.02 (<0.01, 0.12)	0.00 (0.00, 0.00)	0.229
Other DSM axis I disorders	0.00 (0.00, 0.00)	0.06 (0.03, 0.11)	0.130	0.00 (0.00, 0.00)	0.06 (0.03, 0.11)	0.130
Any mental disorder (excluding dementia)	19.06 (17.97, 20.20)	14.93 (14.39, 15.49)	<0.001	13.43 (12.49, 14.42)	11.87 (11.38, 12.38)	0.004

*CI, Confidence interval; NOS, Not otherwise specified; GMC, General medical condition.*

* *: Only have a current diagnosis.*

† *: Cannabis, stimulant, opioid, cocaine, hallucinogen, multiple drugs, and other substance use disorders;*

‡ *: Anorexia nervosa, bulimia nervosa, and binge eating disorders*.

**Figure 2:**
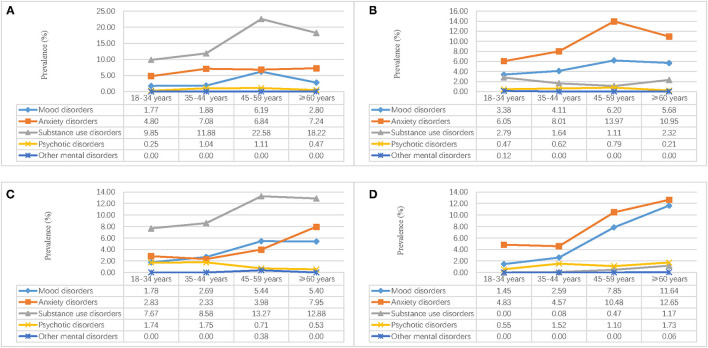
Weighted region-gender-age-specific lifetime prevalence of mental disorders in Hebei province, China. **(A)** Urban male; **(B)** Urban female; **(C)** Rural male; **(D)** Rural female.

**Figure 3:**
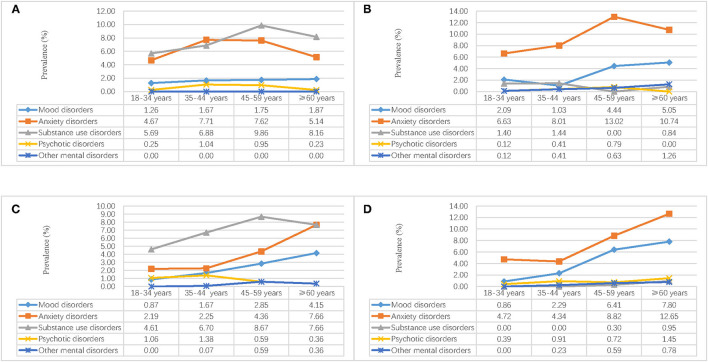
Weighted region-gender-age-specific 1-month prevalence of mental disorders in Hebei province, China. **(A)** Urban male; **(B)** Urban female; **(C)** Rural male; **(D)** Rural female.

In Figures 2, 3, we calculated the weighted lifetime and 1-month region–gender–age-specific prevalence of mental disorders. The results implied that substance use disorders in men and anxiety disorders in women were the most serious problems in Hebei province. In the rural region, the prevalence of anxiety increased rapidly after people aged 35 years. The prevalence of mood disorders was higher in the urban region and increased with age. For psychotic disorders, the prevalence in rural males aged 18–44 years and rural females aged 60+ was higher.

Finally, we analyzed the comorbidity of the weighted lifetime and 1-month diagnoses of mental disorders in Table 7. For lifetime diagnoses, the results showed that there were 491 (2.35%) subjects who met the diagnoses criteria for two, and 76 (0.36%) subjects met the diagnoses criteria for three or more. Similarly, 344 (1.65%) subjects met the diagnoses criteria for two, and 71 (0.34%) subjects met the diagnoses criteria for three or more for the1-month diagnoses.

**Table 7:** Comorbidity of the weighted lifetime and 1-month diagnoses of mental disorders [*n* (%)].

**Number of diagnoses**	**Weighted lifetime diagnoses**	**Weighted 1-monthdiagnoses**
0	17,569 (84.13)	18,331 (87.78)
1	2,749 (13.16)	2,138 (10.24)
2	491 (2.35)	344 (1.65)
≥3	76 (0.36)	71 (0.34)
Total	20,884 (100.00)	20,884 (100.00)

## Discussion

The current study was conducted in Hebei province, an economically underdeveloped region in China, to get the lifetime and 1-month prevalence of mental disorders. The results found that the lifetime prevalence of mental disorders was 15.87% (95% CI: 15.38–16.38%) and the 1-month prevalence of mental disorders was 10.79% (95% CI: 10.37–11.22%). Comparing with the Chinese studies, it is similar to the results in CMHS (16.6%, 9.3%) (20), but it is higher than the results in TJMHS (23.6%, 12.8%) (21). The reason may be explained by regional differences. The current study was interviewed in an underdeveloped province, but TJMHS was implemented in metropolitan China. People who lived in metropolitan had a higher level of life and work stress, which are risk factors for mental disorders (35).

However, when we compared the findings with many other Western countries, it is much lower. According to the WHO's World Mental Health Survey, the lifetime prevalence of mental disorders ranged from 12.0% in Nigeria to 47.4% in the USA, and most of the available data are higher than the prevalence in the current study (36). In the NCS-R study in the USA, the lifetime prevalence of mental disorders was 26.2% in 2001–2002 (37), and it is higher than the prevalence in the current study. The Global Burden of Disease (GBD) 2017 showed that 11,46,401.2 thousand people were with mental disorders and substance use disorders. They mean that the prevalence of mental disorders was about 15%. This data was similar to our findings (16.6%).

In the current study, we also found that the prevalence of anxiety disorders was highest in all of the classifications of mental disorders. It is consistent with recent Chinese studies in CMHS (20), TJMHS (21). However, in the last decades, a study in four Chinese provinces supported mood disorders rank first in the classification of mental disorders (19). In recent years, China is one of the few countries with high-speed development in the world, and Chinese society changes very fast. People need to adapt to the changing society, which is also a risk factor for anxiety disorders (38). This may be a reason for changing the order of anxiety and mood disorders.

The prevalence of some diagnoses with NOS is at a high level in the results, such as anxiety NOS, depressive disorder NOS. This may be caused by the characteristics of mental disorders NOS. Sometimes, we can also see them as the “subthreshold disorders (39, 40),” but they are also at a higher risk of developing full-down psychiatric disorders (41). Thus, the findings imply to us that there are many residents at high risk of full-down psychiatric disorders, and we need to pay more attention and work on them to control the development of mental disorders.

Gender differences were also supported for other kinds of mental disorders, and the prevalence for men is higher than for women. It was mainly caused by substance use disorders, and the other kinds of mental disorders were higher for women. The results align with the previous studies in China and other countries (42, 43). In China, the differences in the lifetime prevalence of substance use disorders are larger than most of the other countries (11.61 vs. 0.79%), and there are more male alcohol drinkers than females. The gap is also very big, especially in the north of China (44, 45). One of the reasons is the Confucian culture in China. The gender differences for other kinds of mental disorders, it may be caused by the sex hormone (46, 47).

We also found that mood and anxiety disorders increased with age, but substance use and psychotic disorders were higher in residents aged 35–44 years. In the rural region, mood disorders and psychotic disorders were higher, and anxiety disorders and substance use disorders were higher in the urban region. Similar findings were also found for the 1-month prevalence of mental disorders expect for substance use disorders. All of these findings were consistent with previous studies in China and other countries (7, 20, 48). Comparing with the rural region, people who live in the urban region have heavier work-load and life stress (49, 50), and they may be at higher risk of mental disorders. Substance use disorders, one way to deal with stress, maybe also in higher-level for rural residents. However, for rural residents, most of them were lower educated, and they were lack of coping skills, which are also risk factors for mood disorders and psychotic disorders (51, 52).

We also analyzed the comorbidity of the diagnoses for the mental disorders, and the results showed that most of the residents were diagnosed with one mental disorder (13.16% for lifetime prevalence and 10.4% for 1-month prevalence). Few residences can be diagnosed with more than two kinds of mental disorders (2.71% for lifetime prevalence and 1.99% for 1-month prevalence). They are lower than the percentages found from the NCSR (3.8%) (37). As we discussed before, the prevalence of mental disorders was lower than in other Western countries, and the severity of mental disorders was not at a high level. Thus, the comorbidity of the diagnoses for the mental disorders was lower than findings from other studies.

There are some limitations, which should be considered when we interpret the findings. First, the sample in the current were community residents aged 18 years and older, and people aged lower than 18 years and who lived in construction sites, armed services, schools, and hospitals were not included in the design. Second, all of the diagnoses were from SCID axis I, and the Axis II diagnoses and dementia were not reported in the current study. Third, the sample size was calculated by the prevalence of schizophrenia, and the prevalence of some mental disorders with lower prevalence is not confident in the current study. Finally, the time frame for the current prevalence was 1 month, and the results cannot be compared with others with different time frames.

Despite these limitations, this is the first large-scale mental health epidemiological study, which conducted in a Chinese economically underdeveloped province using SCID diagnoses. The results are more confident compared with other studies in China in the recent 10 years because of the instruments and strict quality control. The results also can help us to allocate health resources and set up interventions.

## Data Availability Statement

The raw data supporting the conclusions of this article will be made available by the authors, without undue reservation.

## Ethics Statement

The studies involving human participants were reviewed and approved by The Human Research Ethics Committee of Hebei Mental Health Center. The patients/participants provided their written informed consent to participate in this study.

## Author Contributions

LS analyzed the data and wrote the manuscript. YZ was a major contributor in writing the manuscript. KL designed this study and gave important comments on the manuscript. LC, JL, LL, XS, YL, and LZ collected the data. All authors read and approved the final manuscript.

## Funding

This research was supported by the National Natural Science Foundation of China (71603149 and 71974114). The funders had no role in the study design, data collection and analysis, writing the paper and the decision to submit the paper for publication.

## Conflict of Interest

The authors declare that the research was conducted in the absence of any commercial or financial relationships that could be construed as a potential conflict of interest.

## Publisher's Note

All claims expressed in this article are solely those of the authors and do not necessarily represent those of their affiliated organizations, or those of the publisher, the editors and the reviewers. Any product that may be evaluated in this article, or claim that may be made by its manufacturer, is not guaranteed or endorsed by the publisher.

## Abbreviations

WHO: World Health Organization
WMHS: World Mental Health Survey
USA: United State of America
GHQ: General Health Questionnaire
SCID: Structured Clinical Interview for Diagnostic and Statistical Manual (DSM-IV) Axis I disorders
CI: Confidence Interval
NOS: Not Otherwise Specified
GMC: General Medical Condition.
